# Clinically Meaningful Use of Blood Tumor Markers in Oncology

**DOI:** 10.1155/2016/9795269

**Published:** 2016-11-30

**Authors:** Stefan Holdenrieder, Lance Pagliaro, David Morgenstern, Farshid Dayyani

**Affiliations:** ^1^University of Bonn, Bonn, Germany; ^2^Mayo Clinic, Department of Oncology, Division of Medical Oncology, Rochester, MN, USA; ^3^Roche Diagnostics, Indianapolis, USA; ^4^Roche Diagnostics International, Rotkreuz, Switzerland

## Abstract

Before the introduction of modern imaging techniques and the recent developments in molecular diagnosis, tumor markers (TMs) were among the few available diagnostic tools for the management of cancer patients. Easily obtained from serum or plasma samples, TMs are minimally invasive and convenient, and the associated costs are low. Single TMs were traditionally used but these have come under scrutiny due to their low sensitivity and specificity when used, for example, in a screening setting. However, recent research has shown superior performance using a combination of multiple TMs as a panel for assessment, or as part of validated algorithms that also incorporate other clinical factors. In addition, newer TMs have been discovered that have an increased sensitivity and specificity profile for defined malignancies. The aim of this review is to provide a concise overview of the appropriate uses of both traditional and newer TMs and their roles in diagnosis, prognosis, and the monitoring of patients in current clinical practice. We also look at the future direction of TMs and their integration with other diagnostic modalities and other emerging serum based biomarkers, such as circulating nucleic acids, to ultimately advance diagnostic performance and improve patient management.

## 1. Introduction

The term tumor marker (TM) traditionally has referred to substances, mainly proteins, that are either directly produced by malignant cells or are produced by other cells, in response to certain malignant or other nonmalignant conditions. TMs can be associated with malignancies of a specific organ (e.g., prostate surface antigen [PSA] in prostate cancer and thyroglobulin in thyroid cancer), but often a TM, such as cancer antigen 19-9 (CA 19-9), can be elevated in a variety of cancers (e.g., pancreatic cancer, hepatobiliary cancers, and gastric adenocarcinomas) [1]. In addition, TMs are not uniformly elevated in all patients diagnosed with a specific malignancy (e.g., carcinoembryonic antigen [CEA] in colorectal carcinoma [CRC]) [2]. Despite these limitations and prior to the advent of modern imaging techniques and advances in molecular diagnosis, TMs were among the few available diagnostic tools for management of oncologic patients. They are easily measured in bodily fluids, mainly in serum or plasma samples; the results are rapidly available, and the associated costs for TM testing are relatively low [3]. Thus, for many malignancies, TMs have become an established part of patient management and are also included in a number of clinical guidelines [4–11]. The lack of diagnostic alternatives and poor treatment options for patients with advanced cancers highlighted the need for early detection and led the medical community to conduct several studies that tested single TMs for the screening of several solid tumors. However, the various causes of their elevation in blood were associated with insufficient sensitivity and specificity in asymptomatic patients, thus making the use of a single TM for screening in the majority of solid tumors extremely challenging. Even in rare exceptions, such as prostate cancer, where a specific TM, namely PSA, was initially recommended for screening, the intended use of the marker has more recently come under scrutiny because PSA alone cannot distinguish the presence of clinically relevant forms of aggressive cancer from more indolent variants of the disease and thus has led to overdiagnosis and overtreatment [12]. Nevertheless, in case of suspicious masses, studies have shown that newer TMs provide improved profiles of sensitivity and specificity for defined malignancies such as progastrin-releasing peptide (ProGRP) for small cell lung cancer [13] and human epididymis protein 4 (HE4) for ovarian cancer [14].

TMs were discovered in an era prior to the advent of evidence-based, guideline-driven medicine, and many studies examining the utility of TMs were either underpowered, were used to correlate TM levels with what are now outdated “gold standards” (such as plain X-rays to assess tumor response), or did not show the rigorous design required nowadays to conclusively demonstrate a clinically useful endpoint [15, 16].

Over the past decade, advances in molecular and cellular biology have led to the introduction of novel diagnostic tools in oncology which measure circulating tumor cells or elucidate the molecular events of tumors on a single patient level, leading to a paradigm shift in how antitumor therapies are developed and patients are selected for specific targeted therapies [17–19]. The intense focus on the characterization of tumor tissue over the past decade, using gene arrays, polymerase chain reaction (PCR), fluorescence in situ hybridization (FISH), immunohistochemistry (IHC), and next generation sequencing (NGS), has transformed oncology and made precision medicine a reality for many patients [17]. Only very recently we have been able to measure total and mutated cell-free nucleic acid, specific to the patient's tumor, in peripheral blood, which will open up vast new opportunities for diagnosis and treatment decisions for the near future [20–27].

Given the exciting advances in molecular diagnostics, the question arises: will there be any future role for traditional TMs in oncology? While studies on molecular markers in tumor tissue and blood already show great progress in the establishment and validation of new technologies, many inherent biological limitations are still present, including the heterogeneity of the tumors, the prevalence of tumor-specific mutations only in subgroups of cancer patients, and the heterogeneous responses to targeted therapies in the same (e.g., mutation-positive) patient subgroups [28].

We therefore suggest that the optimal patient management flow of the future will integrate novel and established tools, including TMs, and it will be crucial to choose the right marker in the right setting, not only to optimize patient-level outcomes but also to contain associated health care costs to the society as a whole.

The primary goal of this concise review is to conceptually outline how “traditional” TMs can still be clinically valuable.

## 2. Role of TMs in Current Clinical Practice

Despite the diagnostic advances in oncology, the use of TM in the management of patients with solid tumors has several established indications as well as opportunities for broader use. In this section, we will briefly summarize how TMs can be used at each step along the entire spectrum of patient management. The focus here is not an in-depth discussion of novel biomarkers such as circulating tumor cells, circulating nucleic acids, or novel proteomics approaches, since this would go beyond the scope of this overview. However, the authors will define a continued role for TMs in the context of the growing importance of novel biomarkers.

### 2.1. Differential Diagnosis

In individuals with a suspected cancer, TMs can be very helpful to narrow down the potential differential diagnoses and to focus the further workup. For example, while carcinoma antigen 125 (CA125) alone has not been recommended for screening for ovarian carcinoma [29], Lokich et al. could show that the combination of CA125 with HE4, using the Risk of Ovarian Malignancy Algorithm (ROMA), might more accurately define the risk of an epithelial ovarian carcinoma, which would prompt a more aggressive diagnostic workup (e.g., laparoscopy) versus a more conservative management strategy of surveillance in low risk women [30]. Furthermore, the recent data on lung cancer screening in high risk individuals using low resolution computed tomography has shown a mortality benefit on a population basis [31]. However, many intermediate size pulmonary nodules evade a clear diagnosis based on imaging alone and might be too small for CT-guided or transbronchial biopsy. These patients would currently be followed up with short-term imaging, which is costly and exposes them to additional radiation [32]. A panel of six TMs (CEA, cancer Antigen 15-3 [CA15-3], squamous cell carcinoma antigen [SCCA], cytokeratin 19 fragment [CYFRA 21-1], neuron-specific enolase [NSE], and ProGRP) was recently shown to be more accurate in predicting the presence of lung cancer than either a single TM alone or clinical factors such as tumor size and smoking status [33]. Thus, these TMs could be used for appropriate triage of indeterminate lung nodules. All the more, the pattern of CEA, SCCA, CYFRA 21-1, NSE, and ProGRP has been shown to be very helpful to distinguish between small-cell and non-small-cell lung cancer subtypes (SCLC and NSCLC, resp.) [33]. This is quite relevant considering a significant portion of lung biopsies might be nondiagnostic [34]. Another example for improved diagnostic performance of combining TMs is in the realm of prostate cancer screening. In men with no palpable prostate nodule and a screening PSA value in the so-called grey zone, that is, a PSA between 2 and 10 ng/mL, the clinician cannot reliably distinguish between prostate cancer and benign prostate hyperplasia without an invasive prostate biopsy. In this setting, measuring, in addition to total PSA, the levels of free PSA and calculating their ratio (%fPSA) can be helpful to diagnose underlying prostate cancer [35]. The lower the %fPSA, the higher the probability of prostate cancer. In the seminal study by Catalona et al., testing of %fPSA reduced unnecessary biopsies by 20%, still maintaining a 95% detection rate for prostate cancer [36]. The Stockholm model, which incorporates free PSA with an array of other risk assessment data, was recently shown to be a more sensitive and specific screening test than PSA alone [37].

In patients with diagnosed cancer of unknown primary (CUP), the use of several TMs is part of the diagnostic flow as outlined in guidelines such as National Comprehensive Cancer Network (NCCN) [38]. Depending on which TMs are elevated, a focused diagnostic workup can be directed to certain organs and thus the morbidity, time delay, and cost of further invasive diagnostic procedures (e.g., bronchoscopy) may be reduced. While a tissue diagnosis is almost uniformly required for treatment decisions in oncology, two notable exceptions heavily rely on the presence of TMs. In male patients with a testicular mass and elevated TMs (alpha-fetoprotein [AFP] and human chorionic gonadotropin [HCG], alone or in combination), a biopsy is not needed and orchiectomy is the next therapeutic and diagnostic step. Similarly, in patients with underlying liver cirrhosis, an elevated AFP level, together with characteristic findings on multiphase imaging, is sufficient to establish a diagnosis of hepatocellular carcinoma (HCC) and a biopsy is not recommended.

### 2.2. Staging/Prognosis

TMs are also useful after a diagnosis of a malignancy has been established. While multiple groups have investigated the prognostic role of genomic and immunologic biomarker signatures in early stage NSCLC, Muley et al. demonstrated a relatively straightforward combination of presurgery CEA and CYFRA-21 levels can be prognostic for relapse-free survival in this patient cohort of stage I-IIIA patients [39]. Another recent study demonstrated that, in patients with SCLC, both pretreatment levels and absolute levels of ProGRP at the end of the first chemotherapy cycle are prognostic for overall survival [40]. In primary breast cancers, CA15-3, alone or in combination with other TMs, has been shown to be prognostic in several papers [41–44]. Another example is CA19-9, which can be elevated in hepatobiliary carcinomas, and has long been recognized to be an independent prognostic factor in patients with advanced pancreatic adenocarcinoma and thus has been incorporated as a stratification factor in recent large clinical trials [45, 46].

In clinically organ-confined prostate cancer, PSA of 10–20 ng/mL defines intermediate risk and PSA > 20 ng/mL defines high risk, independent of T classification and Gleason score [47]. In metastatic testicular nonseminomatous germ cell tumors, TM are also used to define good, intermediate, and poor risk patients, independent of tumor sizes and locations [48, 49].

These examples illustrate that TMs can be a valuable tool for prognostication in defined patient cohorts, despite the availability of more elaborate and costly tests.

### 2.3. Treatment Monitoring

The arguably most common and best established use of TMs is disease monitoring during treatment. The association of many TMs with various solid tumors was recognized decades ago, and their use as monitoring markers has become an established component of patient management ever since.  Table 1 shows a list of commonly used TMs with associated malignancies. A more detailed review of TMs with their recommended clinical use according to various guidelines can be found elsewhere [50]. The attractiveness of TMs in disease monitoring is rooted in the basic principle of having a tool which informs the oncologist about treatment success rapidly (at most centers within hours), at a relatively low cost (most TMs cost less than $40 [51]), with minimal inconvenience to the patient (i.e., blood draw versus invasive biopsy or imaging). Also, it is important to note that specificity is less of an issue in patients already diagnosed with a certain malignancy, which further increases the usefulness of TMs in this setting. Finally, a TM such as CA125 can indicate in gastrointestinal and other cancers of the abdominal cavity the presence of omental carcinomatosis, where imaging often fails to detect any measurable disease, and in some cases monitoring of CA125 levels remains the only means to assess treatment success [52].

However, for meaningful clinical interpretation of TM kinetics, the maintenance of the same methods for marker measurement is paramount. Further, additional laboratory parameters such as creatinine, transaminases, and C-reactive protein (CRP) levels are helpful to control potential influencing conditions such as renal and hepatic failure or inflammations. Therapy monitoring can be done most efficiently when blood draws are done at defined time points during treatment, for example, before every application of a new chemotherapeutic cycle, and when biochemical response or progression is done on the basis of defined TM changes in relation to the individual baseline values rather than on fixed cut-off levels like the reference value of any control group. Thereby relevant marker changes can vary considerably due to different half or doubling times of the markers. Although these facts seem to be logical, meaningful changes of TMs are poorly defined and clear schedules of marker determinations are rarely used for response estimation in clinical routine so far.

**Figure 1:**
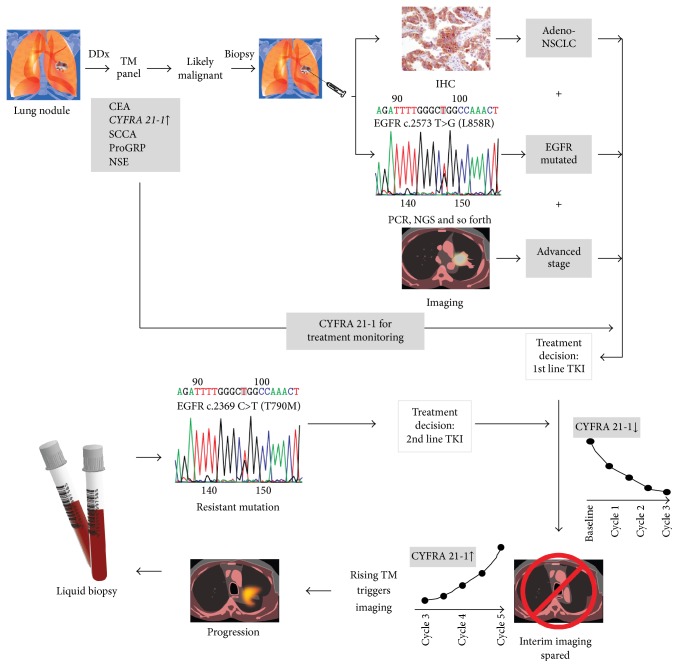
Integration of TMs with other diagnostic modalities, as exemplified in the management of lung cancer. Using a panel of different TMs will guide the decision to either observe a patient with an indeterminate lung nodule versus proceed with a biopsy. In this example, levels of CYFRA 21-1 are elevated. Tissue diagnosis with IHC establishes the diagnosis of adenocarcinoma NSCLC, and molecular testing shows an actionable EGFR mutation. On imaging, an advanced stage is confirmed, and a treatment decision is made based on the integrated information. During treatment (in this case with a tyrosine kinase inhibitor [TKI]), response can be monitored with serial CYFRA 21-1 measurements showing a decline, thus replacing interim staging imaging. Upon rise of the CYFRA 21-1 levels, repeat imaging is performed, which confirms progressive disease. A liquid biopsy avoids an invasive procedure and testing of cell-free DNA by PCR shows the development of a resistant mutation. Based on the result, a second line TKI is chosen. Treatment response is then again monitored using TMs. DDX, differential diagnosis.

The next wave of diagnostic advances in therapy monitoring is focusing on detection of nucleic acids from an individual's tumor in peripheral blood, following the specific mutation in that particular patient [53]. Undoubtedly, this development is another step towards precision medicine and will be an important addition to concept of “treating the right patient with the right medicine.” However, does that mean that TMs will become obsolete in this setting? The answer is very likely no. Molecular diagnostics will be highly valuable at decision points during patient management: at diagnosis to determine the right treatment and at progression to specify the particular resistance mutation (Figure 1). This will dictate the next line of therapy, but* between* decision points, during chemotherapy cycles, it will be very difficult to show a benefit of molecular diagnosis over traditional TMs in disease monitoring. Even today, an early rise in TM might herald future radiographic progression, but might not necessarily lead at the first rise to a change in treatment. Clinical studies comparing circulating tumor DNA (ctDNA) with TMs show ctDNA is best used in patients with nonelevated TM levels [54–57]. No additional benefit has been shown when combined with TMs. This would demonstrate that detecting “molecular progression” is associated with improved outcomes compared to progression based on TMs and thus would justify higher costs and turnaround time. The approach might be useful in selecting highly aggressive cancers with several lines of highly effective therapy, but in the majority of cases this “high sensitivity” method would have a hard time replacing the established TM. In addition, also for molecular markers, clinically meaningful changes have to be defined on a single patient level in order to avoid false positive (or negative) results. Moreover, method continuity has to be considered and preanalytical and influencing factors have to be controlled. This is similar in principle to the introduction of imaging with positron emission tomography (PET) computerised tomography (CT) scan compared to conventional CT scans. For various reasons, including cost, PET scans were not able to routinely replace CT scans during regular disease monitoring intervals. So in the future, while molecular diagnostics will become increasingly important in triaging the patient at decision points, TM in-between those points will continue to be used as a trigger for further elaborate and more costly tests (e.g., imaging or molecular testing for new mutations).

One should not forget that despite fragmentation and subclassification of histologic diagnoses in oncology on a genotypic level to assign the best treatment (e.g., KRAS, NRAS, or BRAF, in mutated CRC [58]), the majority of these are captured under the umbrella of a few blood based TMs on a phenotypic level (e.g., CEA and CA19-9 in CRC). Simply put, that means one biomarker can be used for many molecularly distinct diseases to tell us whether tumor cells are being killed or not. This is clinically relevant as molecular patterns predict treatment response accurately only in a portion of patients while a considerable number (up to 50% in recurrent lung cancer patients) will not respond to targeted therapy approaches, despite positivity of epidermal growth factor receptor (EGFR) mutation analysis [59, 60]. In addition, many patients would not qualify for molecular monitoring as they do not have “drugable” mutations. As molecular profiling shows great interindividual differences, we do not need to create a specific primer set for each patient to monitor their treatment response but can use the same TM for most of them. Thus, disease monitoring during systemic treatment of advanced cancers will remain one of the main indication where TMs will still play an important role in the future.

### 2.4. Surveillance and Recurrence Monitoring

In nonmetastatic cancers which are treated with curative intent and advanced cancers with a good response to first line chemotherapy, surveillance of the patients for up to five years is recommended to detect early recurrence. In some cases, the surveillance time is even longer, for example, in germ-cell tumors [61, 62]. The underlying hypothesis here is that early recurrence detection will increase the likelihood of having a limited disease volume and thus either (a) be able to treat the recurrence with definitive local therapy (surgery and/or radiation) or (b) have a better response to systemic treatment because of smaller tumor load. Hence, in many common cancers that are amenable to screening and thus are detected at nonmetastatic stage in the majority of patients (e.g., prostate, CRC, or breast), the use of TMs in posttreatment surveillance is either already included in the guidelines and/or part of common clinical practice [63–66]. From the above indications to monitor patients for relapse, one can see that usually a salvage treatment, especially a local treatment modality (i.e., surgical or radiation), is based on anatomic localization of the recurrence. Therefore, the TM itself will not trigger the treatment, but rather it will be the next diagnostic test which will ultimately advise on the best treatment strategy. This approach has successfully been applied in breast cancer using CEA and CA 15-3 for after care surveillance [43, 67]. It has to be pointed out that the principles of marker monitoring (maintenance of the same methods, defined time schedules of TM determinations, interpretation according to marker changes in relation to individual baseline values and not according to fixed cut-offs) were the preconditions to develop the most efficient monitoring procedure [68]. Furthermore, triggering sensitive imaging diagnostics and therapeutic interventions were paramount to benefit from the time advantage by early recurrence detection.

Other randomized trials for recurrence monitoring with TMs, for example, in ovarian cancer using CA125, have not shown a survival benefit and were not included in recommendations [69, 70]. Several reasons for the disappointing results have been identified such as the unfavorable patient selection with poor prognosis, unsatisfactory surgical results with overly high numbers of tumor-positive margins, interpretation of CA125 levels on the basis of fixed high cut-offs that did not lead to an early recurrence detection, and insufficient second-line treatments [71, 72]. So if detection of recurrence with TMs in these instances is not recommended, then it is difficult to envision even more sophisticated blood markers, such as cell-free DNA, to be widely accepted as surveillance markers considering costs are higher and procedural schedules are not respected with the markers available nowadays.

However, survival might not be the best endpoint to evaluate the role of TMs in this setting. To fully investigate this, it would require large and long-term randomized trials. Given the fact that TMs have been around for decades and already incorporated in patient management, it would be very challenging to find resources to conduct those trials today. Alternative more immediate endpoints to investigate would be the number of surveillance CT scans saved by TM monitoring or the proportion of patients who undergo curative intent salvage treatment. Primrose et al. recently demonstrated in a large prospective trial that CRC recurrence monitoring with CEA alone was not worse than regular CT scans [73]. It is possible to imagine that blood-based molecular tests for tumor recurrence monitoring will compete with TMs in the future, given the probability that they will show a serologic recurrence without any radiographic correlation and hence no actual target for local salvage treatment may result in frequent, expensive repeat testing until a detectable lesion is identified.

## 3. Future Directions

It is clear that TMs can be very useful tools in patient management, if used appropriately. But it is also clear that more work is needed to optimize the clinical use of TMs in daily practice.

### 3.1. Improve Diagnostic Performance

Traditionally, TMs have been used as single markers, which have led to concerns about their low sensitivity or low specificity. This is especially true for tumors that do not overwhelmingly express a specific TM, for example, NSCLC [74]. In this patient population, no single TM is elevated in a large proportion of patients. However, a panel of several TMs will identify in the majority at least one elevated TM, which then could be followed for treatment monitoring. It is important to note that some TMs are correlated with histologic subtype, which can further guide the choice of TM. Other examples include CRC (CA19-9 in addition to CEA [75]) and ovarian cancer (HE4 in CA125 negative tumors [76]). The combination of two or more TMs can also increase the prognostic performance, for example, with CEA and CYFRA 21-1 in NSCLC [39].

Furthermore, in future trials assessing the diagnostic performance of TMs, investigators should not only focus on a single time point but assess the TM trend over a defined period of time, for example, the introduction of TM kinetics using the risk of ovarian cancer algorithm (ROCA) [77] or when assessing the PSA doubling time in patients with prostate cancer [78].

Finally, research is ongoing to find novel TM, which alone or in combination will improve the diagnostic performance in certain indications, for example, the combination of ProGRP, NSE, CYFRA 21-1, and CEA in lung cancer subtyping [33]. In addition, recent data shows that treatment monitoring in patients with SCLC might be optimized using ProGRP and NSE as TMs [40]. Based on the same concept, the addition of nucleic acids (e.g., ctDNA) might help in closing the diagnostic gap in tumors without known TMs (e.g., sarcomas) or in combination improve the monitoring of malignancies with less established TMs (e.g., S-100B in melanomas [79, 80]) Further ongoing novel marker research will hopefully add to our current armamentarium of available TM.

### 3.2. Generate More Robust Data and Educate

Many widely used TM, such as CEA, CA125, and AFP, were described more than 20 years ago, and their adoption into clinical practice preceded the rise of evidence-based medicine [81–83]. Thus, it would be very challenging, if not almost impossible, to find the resources to regenerate clinical data in separate clinical trials to show utility of these TMs. However, there are other opportunities to collect high-quality data to support the use of TMs. One might take already published data and analyze several papers together in form of a meta-analysis, which would provide more evidence for the use of TMs in certain indications. For disease monitoring purposes, it is possible to collect blood samples in a prospective fashion in consecutive patients who are treated for specific tumors with standard therapy outside of a therapeutic clinical trial and correlate the TM changes with documented tumor responses. While this option is certainly less expensive than performing dedicated prospective clinical TM trials, there are always issues with bias, ascertaining TM changes with response and outcome, as well as variable sample collection time points. Hence, we believe the way forward to generate more robust data for existing (and testing novel biomarkers) would be retrospectively in available samples from large and well-annotated clinical trials. As a next step, prospective sample collections could be incorporated in future therapeutic clinical trials. A more rigorous assessment of novel markers in prospective clinical trials that are sufficiently powered and have clear endpoints (e.g., assessment of response by Response Evaluation Criteria In Solid Tumors [RECIST] [84]) will likely increase acceptance of these TMs into clinical practice. This is especially true for less widely used TMs (e.g., HE4), which should be incorporated into future therapeutic trials to demonstrate their utility as a secondary endpoint. These approaches would address many of the issues associated with samples collected from patients outside of trials, for example, the benefit of having a tight time correlation of sample collection with intervention, and clearly defined criteria for response. Finally, the appropriate endpoints to establish the role of TMs in clinical practice should not always be overall survival, as already outlined above. TMs can be used as an inexpensive and fast diagnostic modality to trigger downstream tests, and hence their utility should be tested in the context of health economic studies, that is, change in patient management and savings in treatment and advanced diagnostics-related costs, rather than purely clinical endpoints, such as overall survival.

The use of TMs outside of their already established uses (e.g., germ cell tumors, colorectal, prostate, ovarian, pancreatic, hepatocellular, and neuroendocrine tumors) will also depend on more education of clinicians. This will happen through publications and conference presentations but also should start early during the training of oncology fellows through their supervising senior physicians. Like many other aspects of clinical practice, the use of TMs is likely correlated with the level of exposure to these TMs during medical training.

Table 2 conceptually summarizes how TM collection could be incorporated into future prospective trials. TMs would be drawn at various time points indicated in Table  2(a), with routine imaging and patient followup as specified in the respective protocol. Various calculations using the TM levels at the time points indicated in Table  2(a) would then be carried out according to Table  2(b) to determine the diagnostic performance of the TM for different clinical endpoints. These results would inform a more data driven and rational use of TMs based on the data provided.

## 4. Conclusion

TMs represent a convenient and cost-effective diagnostic tool for the management of various malignancies. Combining several TMs, serial measurements, and incorporation of novel TMs can all improve their diagnostic performance. In evaluating their usefulness, one should understand their role in certain indications (e.g., disease monitoring) as a first-line test to appropriately trigger further workup and more invasive diagnostics, not the TM as a stand-alone test that will directly affect outcomes of the patients. This fundamental conceptual framework will also result in their continued use despite, or as an important complementation to, novel diagnostic modalities such as cell-free DNA testing. Ongoing research and improved future discovery platforms will advance the field of TMs and add novel markers to the already available armamentarium.

## Figures and Tables

**Table 1:** Commonly used TMs and associated malignancies.

TM	Type of malignancy	Differential diagnosis	Prognosis/staging	Treatment monitoring/surveillance
Tg	Thyroid	x		x
Calcitonin	Thyroid (medullary)	x		x
*β*2M (beta-2-microglobulin)	Multiple myeloma, CLL		x	
CEA	CRC, pancreatic, gastric/gastroesophageal AC, esophageal AC, NSCLC AC, breast, endometrial, thyroid, c-cell			x
CA 125	Ovarian, breast, omental carcinomatosis	x		x
HE4	Ovarian, NSCLC, endometrial	x		x
Beta-HCG	GCT, choriocarcinoma, urothelial	x	x	x
AFP (alpha-feto protein)	HCC, GCT	x	x	x
CA 15-3	Breast, NSCLC AC		x	x
CA 19-9	Pancreatic, biliary tract, upper GI		x	x
CA 72-4	Upper GI, mucinous ovarian			x
CYFRA 21-1	NSCLC, esophageal, HNSCC, pancreatic, bladder	x		x
S100	Malignant melanoma			x
NSE	SCLC, NET, neuroblastoma	x		x
ProGRP	SCLC, thyroid medullary	x	x	x
Chromogranin A	SCLC, NET	x		x
PSA/free PSA	Prostate	x	x	x
SCCA	Cervix SCC, NSCLC SCC, esophageal SCC, HNSCC	x		
Ig (immunoglobulin)	Multiple myeloma			x
LC (light chains)	Multiple myeloma	x		x
Her-2-neu	Breast cancer			x
TK	Multiple myeloma, CLL	x	x	x

AC, adenocarcinoma; SCC, squamous cell carcinoma; CLL, chronic lymphocytic leukemia; HNSCC, head and neck squamous cell carcinoma; GCT, germ-cell tumor; GI, gastrointestinal; NET, neuroendocrine tumors; TK, thymidine kinase; Tg, thyroglobulin.

**Table:** (a) Schematized prospective trial

	Baseline	Treatment			Surveillance	Progression (PFS)/recurrence (RFS)	Death (OS)
Imaging	T0		T2	T3	T4	T5	
TM	T0	T1	T2	T3	T4	T5	

**Table:** (b) Uses of TMs at different clinical endpoints

Clinical endpoint	Prognostic

Use of clinical data	Correlate T0 levels with PFS/RFS and/or OS
Potential outcome	TM can be used in future trials as prognostic factor for risk stratification
Example for clinical implication	In “good risk patients”: consider less intensive treatment, or shorter duration
	In “poor risk patients”: consider maintenance after induction chemotherapy

Clinical endpoint	Response

Use of clinical data	Correlate change in T0 to T1 and T2 levels with response per RECIST on imaging at T2
Potential outcome	Early TM change at T1 predicts progression on first imaging at T2
	Early TM change at T2 predicts progression on 2nd imaging at T3 (i.e., in patients with stable disease on 1st imaging at T2)
Example for clinical implication	Randomized trial of continuation of same chemotherapy versus early change to different regimen based on early TM stratification; primary outcome could be ORR, PFS/RFS, or OS

Clinical endpoint	Treatment monitoring

Use of clinical data	Correlate change in T0 to T3 levels with best response per RECIST on imaging at T3
Potential outcome	Decline in TM panel correlates with response on imaging
Example for clinical implication	Fewer interval scans for patients with declining markers

Clinical endpoint	Detection of early relapse

Use of clinical data	Correlate change from nadir of TM at T3 with posttreatment at T4 and T5
Potential outcome	Increase in levels of TM at T4 compared to T3 will predict progression at T5
Example for clinical implication	Tailor surveillance imaging based on TM levels

T0 to T5, various time points for blood draw and/or imaging; PFS, progression-free survival; RFS, recurrence-free survival; OS, overall survival.
